# From Decision to Delivery: A Qualitative Study of Non-Institutional Birth Pathways in Northern India

**DOI:** 10.3390/healthcare14183126

**Published:** 2026-09-21

**Authors:** Meghna Singh, Neeraj Sharma, Catherine Arsenault, Wen-Chien Yang, Sasha G. Baumann, Soumya Ranjan Nayak, Emily R. Smith, Sarmila Mazumder

**Affiliations:** 1 Implementation Science Domain, Society for Applied Studies, New Delhi 110016, India; meghna.singh@sas.org.in (M.S.);; 2 Department of Global Health, Milken Institute School of Public Health, George Washington University, Washington, DC 20052, USA; 3 Department of Exercise and Nutrition Science, Milken Institute School of Public Health, George Washington University, Washington, DC 20052, USA

**Keywords:** Non-institutional deliveries, home births, decision making, exploratory qualitative study, India

## Abstract

**Highlights:**

**What are the main findings?**

- The study examined non-institutional deliveries (in-transit and home deliveries), in a setting where access to the health system during the antenatal period is high.
- These non-institutional deliveries were either planned or unplanned, providing insights on decision-making processes concerning the place of delivery and how decisions are made during labour.

**What are the implications of the findings?**

- An elaborated conceptual framework of “decision to delivery pathway” was developed, building on existing continuum-of-care and delay models.
- The conceptual framework identifies modifiable action points contributing to non-institutional delivery to help address the coverage lags in institutional deliveries.

**Abstract:**

**Background:** Determinants of non-institutional delivery, particularly in the antenatal period, have been described previously, but less is known about the sequence of events during pregnancy and decision-making processes occurring between labour onset and birth. **Aim:** We explored reasons for non-institutional births and pathways leading to home and in-transit deliveries in a north Indian setting, with the aim of identifying context-specific, actionable recommendations. **Methods:** Participants were women from the north Indian site of the Redefining Maternal Anemia in Pregnancy and Postpartum (ReMAPP) cohort who experienced non-institutional delivery. We conducted an exploratory qualitative study using a semi-structured in-depth interview guide. An a priori conceptual framework was developed based on existing continuum-of-care literature and structured around the antenatal, intrapartum, and postnatal phases of care. The framework focused on the “decision-to-delivery pathway” and was subsequently iteratively refined and elaborated based on the thematic findings. **Findings:** Of the 2000 women enrolled at the north Indian site of the ReMAPP study, 75 (3.8%) experienced non-institutional delivery; among these, 50 participants were interviewed. Among those interviewed, 19 had planned, and 31 had unplanned non-institutional deliveries. Themes identified for planned non-institutional deliveries included perceived mistrust in healthcare, institutional avoidance, reliance on informal maternity care systems, and decision-making uncertainty. For unplanned non-institutional deliveries, themes included intention to institutional delivery, delay in obtaining appropriate maternity care, physical access barriers, reliance on informal birth assistance, and opportunities to avail health system services for post-delivery or newborn care. **Conclusions:** Our findings suggest that improving institutional delivery coverage requires more than physical access to facilities. Trust, respectful maternity care, timely responsiveness during labour, and birth preparedness appear to strongly influence whether women are able to reach and utilise institutional delivery services.

## 1. Introduction

Institutional delivery is defined as childbirth taking place in healthcare facilities under the supervision of qualified healthcare professionals [1]. It is a critical component in reducing maternal and newborn mortality, which remains a major global health challenge, with most preventable deaths occurring in low- and middle-income countries (LMICs) [2,3,4]. Increasing access to facility-based childbirth and ensuring skilled birth attendance is a central strategy promoted by the World Health Organization to improve maternal and neonatal outcomes [1]. Skilled intrapartum care is critical for the timely identification and management of life-threatening obstetric complications, including hemorrhage, obstructed labour, hypertensive disorders, and sepsis [5,6]. These efforts have contributed to a rise in institutional deliveries globally from 62% to 80% over the past two decades [7]. Despite progress, nearly 3 out of 10 births continue to occur outside health facilities and without skilled care, particularly among socially and economically vulnerable populations [7].

India has made substantial investments in the past two decades to promote institutional delivery through various maternal health programs and financing schemes, including Janani Suraksha Yojana (JSY), Surakshit Matritva Aashwasan (SUMAN), Janani Shishu Suraksha Yojana (JSSK) and Labour Room Quality Improvement Initiative (LaQshya) [8,9,10]. These initiatives have contributed to an increase in institutional deliveries from 39% in 2005–2006 to 89% in 2019–2021 [11,12]. However, non-institutional births continue to occur disproportionately among vulnerable populations (lower socioeconomic status, less educated, residing in rural/slum areas), with home birth rates exceeding 30–50% in some of these settings [13,14]. Across Haryana State, institutional delivery coverage exceeds 90%; however, within-state differences persist, with the Palwal district reporting a high proportion of non-institutional delivery (21.7%). Antenatal care coverage also remains suboptimal, with only 53.4% of women completing four or more antenatal visits [15].

Although geographic and financial access to maternity services has improved across India, important gaps remain in the quality, responsiveness, and experience of care. When programmes prioritise institutional delivery targets without equal emphasis on respectful, timely, and trustworthy care, women may disengage from the formal health system or fail to reach facilities in time [16]. Previous studies from LMICs have identified several determinants of non-institutional delivery, including low maternal education, poverty, high parity, rural residence, and inadequate antenatal care utilization [5,17,18,19]. Logistical constraints such as long travel distances, limited transport availability, and concerns regarding out-of-pocket costs or loss of wages further constrain access to facility-based childbirth [6,13,20]. Sociocultural norms and perceptions of care also shape childbirth decisions. In many communities, childbirth is viewed as a natural event that does not require medical intervention unless complications arise, reinforcing the preference for home delivery [13,21]. Traditional birth attendants and family elders are often trusted for their culturally familiar and emotionally supportive care [22,23]. In contrast, past negative experiences within health facilities, including disrespectful treatment, a lack of privacy, informal payments, and limited emotional support from healthcare providers, can discourage women from seeking institutional deliveries [24,25,26].

Existing literature has primarily focused on identifying determinants of non-institutional delivery, particularly in the antenatal period, but less is known about the sequence of events and decision-making processes occurring between labour onset and birth. This intrapartum period is important because decisions are often made under conditions of urgency, uncertainty, and constrained resources. In the Indian context, where institutional delivery is the norm and coverage exceed 90%, the persistence of non-institutional births suggests gaps in evidence to understand why women still deliver outside facilities. This gap is highlighted in our study setting of Palwal, where district-wide institutional delivery coverage lags behind state levels [27,28].

Understanding why women who had regular antenatal care contact and intended facility delivery nonetheless delivered at home or in-transit may reveal critical missed opportunities within the continuum of care. This study aimed to explore the reasons and “decision-to-delivery pathway” leading to non-institutional births (home and in-transit) among women enrolled in a cohort in northern India. Findings are intended to inform context-specific actionable recommendations.

## 2. Methods

### 2.1. Study Design and Setting

We conducted an exploratory qualitative study to gather data through in-depth interviews (IDIs) under the umbrella of the Redefining Maternal Anemia in Pregnancy and Postpartum (ReMAPP) study. The ReMAPP study was a prospective, multisite, open cohort study of pregnant women, aiming to determine hemoglobin cutoffs in pregnancy, including study sites in South Asia and sub-Saharan Africa. The northern India site was in a block of the Palwal district of Haryana [29]. A secondary-level public health care facility was selected as the primary study facility, serving a predominantly semi-rural catchment area, with most households located within a radius of 20 kms. ReMAPP participants at the northern India site were enrolled from the community from December 2023 to March 2025. The study protocol has been published elsewhere [30].

### 2.2. Study Participants

Women enrolled in the northern India site of the ReMAPP cohort who experienced a non-institutional delivery, resided within the study catchment area, and were willing and able to provide informed consent were eligible for inclusion in the present study. The eligible women were identified through cohort surveillance records and contacted by the study team. Interviews were conducted in the postpartum period at participants’ homes at a convenient time while ensuring privacy and confidentiality. Data collection continued until the close of the study period; the final sample therefore reflects participant availability during this window rather than a pre-specified saturation or information-power criterion.

### 2.3. Operational Definitions

Institutional delivery was defined as childbirth (livebirth/stillbirth) occurring in a recognized public or accredited private health facility designated to provide delivery services, with the minimum prescribed infrastructure and skilled personnel required for intrapartum and immediate newborn care according to the Government of India guidelines [11]. Non-institutional delivery was defined as childbirth occurring at home or in transit before reaching a health facility.

The participants were classified under planned or unplanned non-institutional delivery groups, based on the women’s experiences shared during the IDIs. Planned non-institutional delivery referred to the birth in which the woman or her family intended a home delivery before the onset of labour. Unplanned non-institutional delivery referred to the birth in which delivery was intended at a health facility, but childbirth occurred at home or in transit due to any specific or non-specific reasons.

Drivers of non-institutional delivery were referred to as reasons that directly shaped or influenced the decision-making for substitution of formal maternity care. Barriers to institutional delivery were referred to as reasons that prevented, delayed, or disrupted access to facility-based care. Facilitators to institutional delivery were referred to as factors supporting institutional delivery and continuum of care.

The term “decision-to-delivery pathway” in this article is used to describe the sequence of events and decisions occurring during pregnancy through the onset of labour to the point of delivery and immediate postpartum care.

### 2.4. Data Collection Procedure

Data were collected through IDIs using a pretested, semi-structured interview guide between December 2025 and March 2026 (Supplementary File S1). IDIs were conducted by two trained female interviewers with post-graduate education level, prior experience in qualitative research, and fluency in local language (Hindi) and English. Both interviewers were part of the ReMAPP study team. Interviewers may therefore have had prior contact with participants through routine cohort visits, although neither held a direct clinical or gatekeeping role in participants’ care; interviewers explained the distinct, voluntary nature of the qualitative interview at the outset of each visit, and the weekly reflexivity meetings described below specifically discussed instances where a pre-existing rapport with participants may have shaped disclosure, particularly regarding sensitive experiences with healthcare providers. Given the potential sensitivity of childbirth experiences, interviewers were trained to conduct conversations empathetically and to pause or terminate interviews if participants became distressed. Participants were informed about the purpose of questions and objectives of this study prior to data collection. Weekly discussions were conducted with interviewers and study investigators to promote reflexivity, to limit the influence of preconceptions and interpretive biases, and to enhance the transparency and credibility of the analysis.

The semi-structured interview guide was developed based on a literature review and refined through peer feedback and pilot testing in a similar setting before final data collection. It included open-ended questions, with probes on topics to be explored. Interviews were conducted in a manner to capture how decisions unfolded, from the onset and recognition of labour through delivery and the immediate postpartum period. Interviews also explored details regarding antenatal counselling and birth preparedness, intended place of delivery, labour onset and recognition, intra-household decision-making and external influence, transport and referral experiences, perceptions, and prior experiences with health facilities, including respectful care, financial implications, and logistical constraints.

Interviews were conducted in local language and audio-recorded with participants’ prior consent. The interviews lasted 30–45 min on average. Audio files were stored on password-protected devices accessible only to authorized study personnel. Participant identifiers were removed, and unique study identification numbers were assigned to all transcripts and analytic files. Recordings were transcribed in Hindi with the help of field notes by trained, bilingual study staff. The verbatim transcripts were translated into English where required for analysis and manuscript preparation. All translations were cross-checked by bilingual researchers to ensure no contextual meaning was lost. Coding and thematic analysis were conducted using the original transcripts and translated verbatims. Audio recordings were consulted where clarification of meaning, idiom, or nuance was required, including for verbatim quotations reported in the results. Sociodemographic characteristics of participants were obtained from the ReMAPP study database.

### 2.5. Data Analysis Plan

We conducted thematic analysis using a mix of inductive-deductive approaches. A preliminary codebook was developed from the interview guide and existing literature and was iteratively refined as new themes emerged with analysis. Two researchers independently reviewed and coded each transcript in Microsoft Excel; coding was compared, and disagreements in codes or interpretation were resolved through discussion and consensus. The codebook was iteratively added to, merged, and reorganised as each transcript was completed. Similar codes were grouped into categories, from which broader themes were identified. Categories and themes were further refined through collaborative discussions among investigators. We did not undertake participant validation of the categories and themes. Codes were classified as drivers of non-institutional delivery, or as barriers or facilitators of institutional delivery. Using constant comparison, we examined patterns across cases to identify common reasons, sequences of events, and decision-making processes associated with non-institutional deliveries. This study is reported in accordance with the Consolidated Criteria for Reporting Qualitative Research (COREQ) checklist, provided in full as Supplementary File S2.

## 3. Results

**Figure 1:**
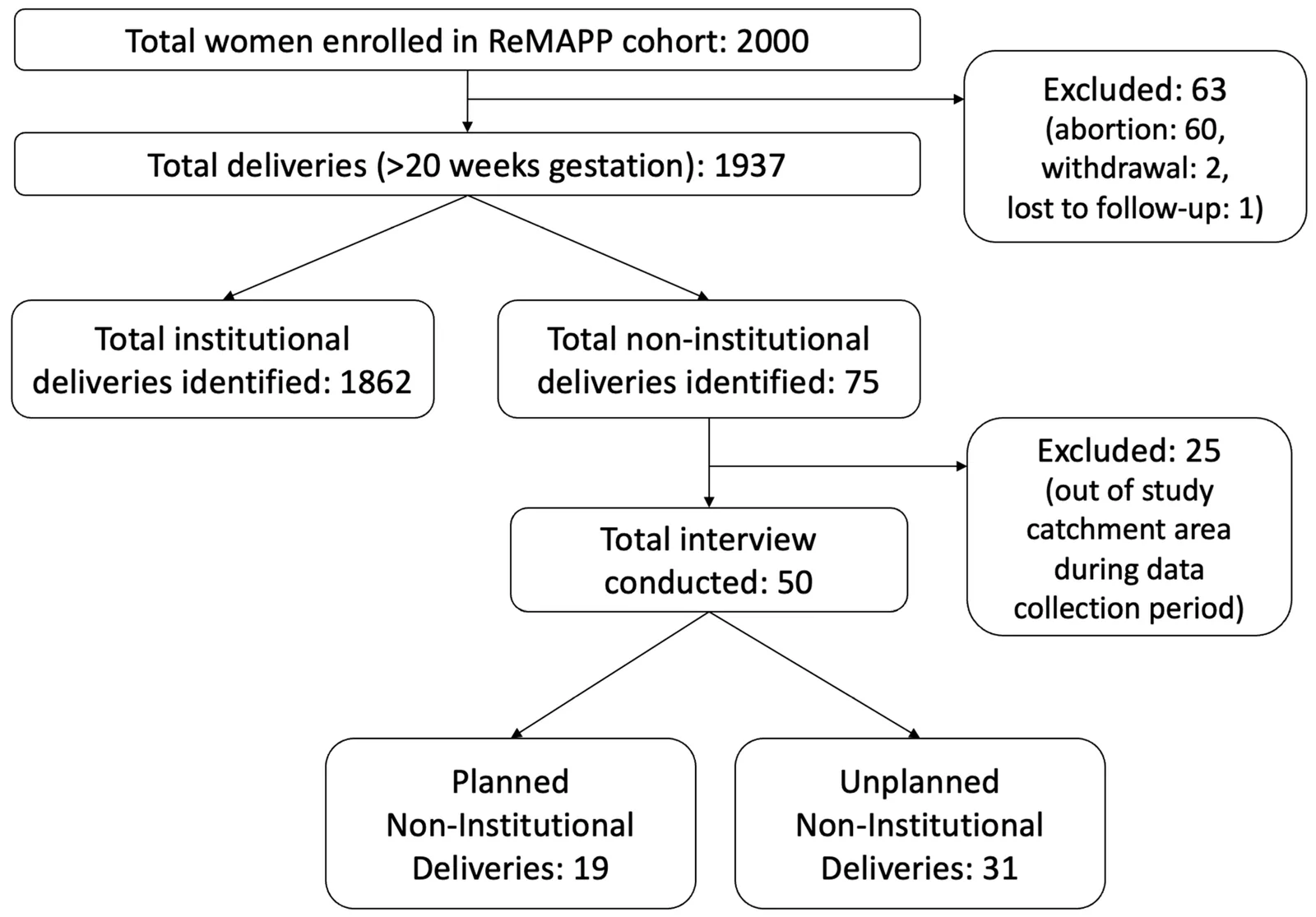
Study flow diagram for participant recruitment. Abbreviation: ReMAPP—Redefining Maternal Anemia in Pregnancy and Postpartum.

Among the 2000 women enrolled in the ReMAPP study, 75 (3.8%) experienced non-institutional delivery, of whom 50 participants were interviewed. The remaining 25 participants were temporarily out of the study catchment area during the data collection period and did not differ in socio-demographic characteristics from the interviewed participants. One interviewed participant declined consent for audio recording. Among interviewed participants, the majority (*n* = 31, 62%) had an unplanned non-institutional delivery and 19 (38%) had planned to give birth at home (Figure 1). Five stillbirths were reported; all stillbirths were unplanned non-institutional deliveries. Most participants were aged 18–24 years, had completed more than 9 years of schooling, were primarily homemakers, multiparous, and the majority attended four or more antenatal care visits. Decisions regarding the place of delivery were primarily made by the husband or mother-in-law, with greater than 70% in both planned and unplanned non-institutional delivery groups (Table 1). The median interval between delivery and interview was ten months, ranging from 2 to 18 months, and did not differ substantially between the planned and unplanned pathways (12 v/s 10 months).

### 3.1. Conceptual Framework of “Decision-to-Delivery Pathway”

**Figure 2:**
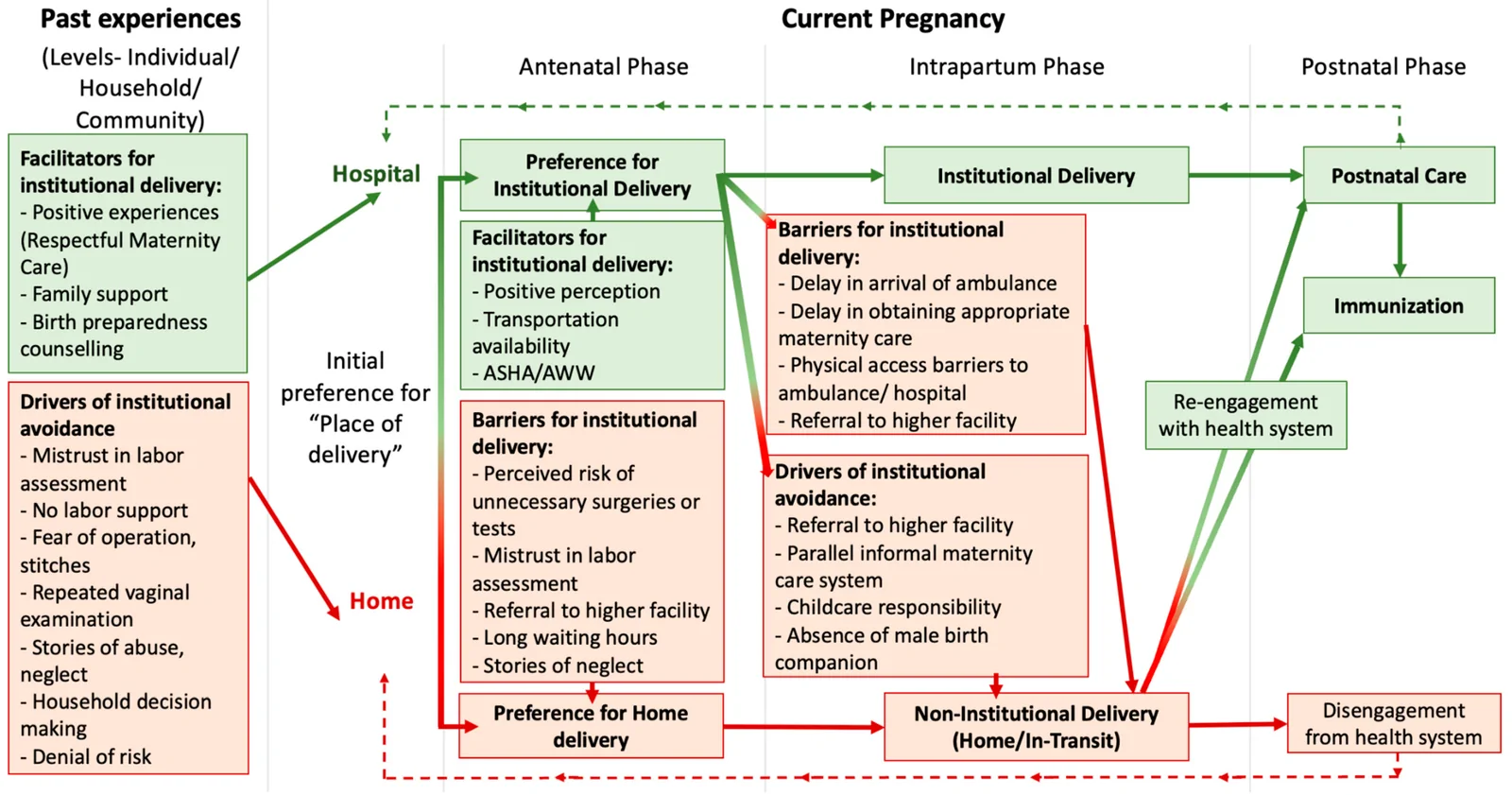
Conceptual framework of *“decision-to-delivery pathway”* and modifiable points contributing to non-institutional delivery. The figure illustrates how women’s preferences to seek institutional delivery care change during the antenatal, intrapartum, and postnatal phases, and are based on the learnings from their past experiences. Green arrows represent the continuum of care, which is supported by the facilitators for institutional delivery (green rectangular box). Red arrows represent the planned non-institutional deliveries. Drivers of non-institutional delivery (red rectangular boxes) are referred to as reasons, which directly shaped/influenced the decision-making for substitution of formal maternity care. Barriers to institutional delivery (red rectangular boxes) are referred to as reasons that prevented, delayed, or disrupted access to facility-based care. The framework organises identified themes within a chronological structure defined a priori; it summarises recurring patterns across participants’ accounts and does not represent empirically tracked, case-level sequences of decision-making for individual women. Abbreviations: ASHA—Accredited Social Health Activist, AWW—Anganwadi Worker.

Based on the information captured during the interviews and informed by existing literature, we developed a visual conceptual framework of “decision-to-delivery pathway” (Figure 2). This framework’s broad chronological structure (antenatal, intrapartum, and postnatal phases) was defined a priori to guide data collection; the specific facilitators, barriers, and drivers populating each phase were derived inductively from the thematic analysis described in Section 2.5, and the framework is a conceptual synthesis of reported experiences rather than an empirically validated, case-tracked sequence of decisions for individual women. The pathway illustrates a common process for how women’s initial delivery preferences may be shaped by various facilitators and barriers, influencing healthcare-seeking behaviour across different phases of pregnancy, from the antenatal, intrapartum, through the postnatal period, which highlights several modifiable action points. Women’s decisions are often driven by their experiences in past pregnancies, where positive encounters act as facilitators for institutional delivery in the current pregnancy and negative experiences such as poor quality of care, failed referrals, or abuse act as drivers for institutional avoidance. During the antenatal phase, initial preference for institutional or home delivery is influenced by her active engagement with the healthcare system and her positive or negative experiences. In the intrapartum period, during the active phase of labour and delivery, women face barriers in accessing the healthcare system due to delays in the arrival of an ambulance, a delay in obtaining appropriate maternity care, physical access barriers to an ambulance or hospital, or referral to a higher facility, leading to unplanned non-institutional deliveries. However, some community and household drivers in this phase, such as the influence of a trusted informal care provider (Dai) or the denial of risk by family elders, create an impact that shifts women toward non-institutional delivery by choice. In the immediate postnatal period, some women seek the opportunity to actively engage with the healthcare system for post-delivery maternity and newborn care, including immunisation. Yet, many others disengage from the health system following non-institutional delivery.

### 3.2. Thematic Analysis Findings

The thematic analysis revealed two distinct pathways: planned and unplanned non-institutional deliveries. Four major themes under planned non-institutional delivery were: (1) perceived mistrust, (2) institutional avoidance driven by fear and negative narratives, (3) parallel informal maternity care systems, and (4) negotiated decision-making within the household. Five major themes identified under unplanned non-institutional deliveries were: (1) intention for institutional delivery and preparedness, (2) delay in obtaining appropriate maternity care, (3) physical access barriers, (4) seeking informal birth assistance in emergent situations, and (5) opportunities to avail health system services.

### 3.3. Planned Non-Institutional Deliveries

A total of 38% (19/50) participants had a planned non-institutional delivery, characterized as an intentional and progressively constructed decision to deliver outside of a health facility. The “decision-to-delivery pathway” among these women was primarily motivated by a mistrust of the healthcare system. Women instead reported relying on the opinions of household elders and/or Dais (i.e., traditional birth attendants), at times denying the risk of potential medical complications inherent to non-institutional deliveries (Table 2).

#### 3.3.1. Perceived Mistrust

A common starting point was a profound lack of trust in healthcare providers and institutional maternity services from their own or family members’ perceptions. Many participants described experiences in which labour was either inadequately assessed or the provider missed the correct diagnosis, with women being advised to return home despite being in active labour. Several women subsequently delivered soon thereafter with assistance of Dais, reinforcing their perception of incompetence within the formal healthcare system. Some women reported receiving inadequate attention or support during labour in their prior pregnancies. A participant said: “We went to the hospital, but no one gave any injection to decrease the pain. I went at the time of all three children, no one gave an injection... I did go this time, but they didn’t pay attention to me, they didn’t help me in the hospital, I went twice, both times they did the same thing (ID 19, planned).” Participants also perceived that hospitals often recommended procedures, referrals, or tests unnecessarily, often imposing additional financial burden. Collectively, these experiences contributed to a broader perception that institutional maternity care was unreliable and unsupportive.

#### 3.3.2. Institutional Avoidance Driven by Fear and Negative Narratives

Fear emerged as a significant deterrent to seeking institutional care, particularly fears surrounding surgical intervention, suturing, and repeated clinical examinations. These apprehensions were shaped not only by women’s personal experiences but also by stories circulating within the community. A woman narrated her experience “In my previous delivery, everyone, by putting their hands again and again, kept saying that the mouth (cervical opening) is not open... I have thought that I will not go, the madam, you know, she also put her hand below, checked and wrote on the paper, did not even tell me... I feel scared... that they check again and again (ID 17, planned).”

Participants reiterated experiences of long waiting times, verbal reprimand and physical mistreatment, all of which influenced their healthcare-seeking decision. Consequently, hospitals were often perceived as spaces that generated more stress and humiliation rather than reassurance and care. A participant said, “Madam (doctor) shouts a lot, I am very scared… that at the time of delivery, everyone shouts, whoever goes for delivery tells... that if they cry, then they are beaten... they (staff) also hit on the cheeks, and also hit on legs... so I am scared (ID 19, planned).”

#### 3.3.3. Parallel Informal Maternity Care Systems

In contrast to the perceived risks with institutional care, a parallel system of maternity care primarily centred around Dais emerged as a trusted and accessible alternative. Dais were readily available within the community, often residing in close proximity to families, and frequently served as the first contact point during pregnancy and labour. Participants described a strong sense of familiarity, comfort, and trust in their experiential knowledge and support. One participant said, “The Dais in that place are very skilled in this (conducting deliveries), whatever she says, she does, if she says she will deliver the baby within three hours, she delivers within three hours. This is a confirmed assurance (ID 29, planned).”

Practices typically considered unsafe within formal healthcare settings, such as unskilled providers managing complicated deliveries without adequate equipment, administering injections without formal training, or conducting deliveries in unsterile environments, were often normalised and accepted by participants and their families. As said by a participant, “She (Dai) has conducted many deliveries, even in cases where the hospital said it would require an operation, she managed it and got it done as a normal delivery (ID 29, planned).” The assurance of a “normal delivery” offered by Dais, coupled with flexibility to negotiate costs, contributed to acceptability and reliance on this mode of care among many financially deprived families.

#### 3.3.4. Negotiated Decision-Making Within the Household

Decisions regarding place of delivery and healthcare seeking were rarely made by the woman alone. Husbands and senior family members, mainly mothers-in-law, exhibited substantial influence over decision-making. In several instances, family members actively discouraged institutional delivery, drawing upon prior negative experiences, collective memory, and perceived risks with facility-based care. An underlying sub-theme across this pathway was the normalization of home delivery. Childbirth was commonly perceived as a natural event that did not necessitate medical intervention unless any complications emerged. The home environment was frequently described as safe, familiar, and culturally appropriate, thereby diminishing the perceived need for institutional care. As one participant shared, “In the village, everyone gives birth at home, only when there is a big problem, or issue, then they visit the hospital… In the village, everyone gives birth at home, even now they are doing it… only when there is a big problem or the child is not coming, then they go (ID 13, planned).”

For some participants, competing domestic responsibilities further constrained access to health facilities. Childcare obligations, limited intra-household support, and the absence of an available male companion for transportation emerged as additional barriers. One participant said, “We need someone at home too, I had to take care of the children at home, I don’t have a mother-in-law. Earlier (in the previous pregnancy) my sister took care, when I went to have a baby. Now she too is busy working (ID 42, planned).” Although these factors alone may not have determined the place of delivery, they interacted with mistrust in institutional care and preference for informal or home-based care, ultimately reinforcing the decision for home delivery.

### 3.4. Unplanned Non-Institutional Deliveries

Overall, 62% (31/50) of women in the sample had an unplanned non-institutional delivery, inclusive of births that occurred outside of the health facility despite a primary desire for institutional delivery. These women were often hindered by transportation and infrastructure challenges, along with inaccurate labour timing, either due to a lack of judgement or other circumstances. The “decision-to-delivery pathway” for unplanned non-institutional delivery involved an initial intention to seek care in a facility despite logistical constraints during labour. These barriers contributed to reliance on informal birth assistance, and shaped participants’ and their family members’ decision for an unplanned non-institutional delivery. Some of these participants subsequently re-engaged with the health system for post-delivery or newborn care (Table 3).

#### 3.4.1. Intention for Institutional Delivery and Preparedness

These participants presented a preference for institutional delivery, arising from prior positive experiences and recognition of the benefits of facility-based care. A participant said, “All the facilities are available in the hospital. All the children in this house were born in the hospital only. Why should it be done at home, do you see any facilities at home? I don’t see any, the children do well in the hospital, as they have the facilities for everything (ID 26, unplanned).” Many participants had made arrangements for transportation, either through their own vehicle, ambulance services, or with the support of local Accredited Social Health Activist (ASHA) or Anganwadi workers (AWW).

#### 3.4.2. Delay in Obtaining Appropriate Maternity Care

In unplanned deliveries, a critical disruption in the pathway to institutional delivery occurred at the onset of labour. While some participants were unable to recognize labour signs due to their first experience, several participants reported being discharged or sent back home after being informed by a healthcare provider at a facility that labour had not progressed sufficiently or due to false labour. However, the women delivered at home within a short period of time after returning home. A participant narrated, “We went to the hospital in the daytime, they sent me back, on Monday night, a girl was delivered at midnight. I came and lay down on the cot (bed), the child delivered there itself (ID 03, unplanned).”

#### 3.4.3. Physical Access Barriers

Participants described external infrastructural barriers, including railway crossings, road closures, poor road conditions, and household mobility constraints, that delayed access to ambulances and healthcare facilities. A participant reported, “We called the hospital for ambulance, the ambulance arrived, but there was a roadblock at railway crossing, he (ambulance driver) told I will not be able to come, you come by car here, but we had no one here, we told him that we won’t be able to come till there, he returned from there... the road remained closed for 2–3 months, a bridge was being built... there is no other way... the road is broken (ID 15, unplanned).”

#### 3.4.4. Seeking Informal Birth Assistance in Emergent Situation

For some women, delivery occurred in an emergent manner. In these cases, assistance was sought from whoever was available, including Dais, family members, or nearby individuals without prior delivery experience. Unlike planned non-institutional deliveries, these circumstances were characterized by inadequate preparedness and heightened uncertainty, reflecting systemic failures at critical points rather than a deliberate preference for non-institutional deliveries. A participant said, “We called the ambulance, the ambulance came and stopped, but before that the child was born, so the ambulance went empty, it was raining heavily….. There is a girl living in the neighbourhood, her mother used to do deliveries, we called her for help, she cut the cord (ID 23, unplanned).”

#### 3.4.5. Opportunities to Avail Health System Services

Despite the break in the care continuum around the time of delivery, participants often accessed postpartum or newborn care within institutions following birth. This included emergency obstetric responses after non-institutional deliveries, postnatal visits for maternal or neonatal wellbeing, and immunization services. A participant narrated, “At first a girl child was born… as soon as she fell in the toilet, I quickly caught her, after catching her, her umbilical cord came out, then we called our sister, so she called the ambulance, the ambulance came and took us, girl was born at home, then a boy was born in the government hospital... (ID 07, unplanned).”

## 4. Discussion

In this study, we explored non-institutional births among women enrolled in a pregnancy cohort in Palwal, India using a “decision-to-delivery pathway,” that traces how intentions and decisions evolve from the antenatal period through labour and the immediate postpartum.

Three features distinguish this work from existing evidence. First, we studied non-institutional birth in a setting where institutional delivery is already the norm, and where residual non-institutional births likely reflect a complex interaction of individual, household, sociocultural, access, and health systems, rather than a single dominant driver such as unmet demand for facility care. Second, all participants were engaged with the health system: 88% had completed four or more antenatal visits, and all were under active cohort surveillance. These are women the system had already reached. Third, and most consequentially, the majority of non-institutional births in this cohort were unplanned, occurring among women who intended, and had prepared for, facility delivery. Prior qualitative studies have documented why women prefer home birth [16,22,25,26]; far fewer have reconstructed how women who wanted facility birth nonetheless delivered at home or in transit. Treating non-institutional delivery as a single outcome, as most survey-based studies do, conflates two populations whose, decision timelines, and policy remedies differ substantially [2,7,31,32]. The present study identified reasons for these outcomes not previously well characterised in this setting, defined within two distinct pathways: planned and unplanned non-institutional deliveries.

We found that planned non-institutional deliveries were primarily driven by mistrust in the health facility (including providers’ clinical judgement), fear of medical interventions, social influence, and preference for informal or home-based care. Global evidence aligns with these findings; medicalisation of childbirth, fear of surgical procedures, and preference for traditional birth attendants have been shown to influence women’s decisions away from formal health facilities [16,22]. Similar findings are reported in qualitative studies from India that emphasise that lack of respectful maternity care, previous experiences of abuse and neglect, denial of obstetric risk, and decision-making by household elders further shape deliberate institutional avoidance [23,25,33]. Trust in the health system appeared to be a central theme in healthcare-seeking behaviour. This is similar to other studies, where women and families were more likely to engage with institutional care when services were perceived as accessible, responsive, and supportive [16,19,22,34]. Repeated interactions with healthcare providers during antenatal care appeared to strengthen confidence in the formal health system and may explain the lower proportion of non-institutional deliveries observed in this cohort compared with district-level estimates. These findings reinforce the importance of continuity of care and perceived responsiveness in shaping delivery decisions [19,22,35].

In contrast, we found that unplanned non-institutional deliveries were largely driven by breakdowns in access, transport, and health-system responsiveness. Literature exploring the reasons among women who intended to deliver in health facilities identified challenges commonly occurring during labour recognition, transport mobilisation, and interactions with healthcare facilities [36,37]. A frequently reported finding observed in the present study involved women being sent back home because labour was considered insufficiently advanced, followed by rapid labour progression and subsequent home or in-transit delivery [20,38,39]. These findings highlight the need to strengthen responsiveness and support during the intrapartum period, as several barriers contribute to unplanned non-institutional deliveries. In particular, women being turned away during labour raises concerns regarding the assessment and management of women presenting in labour, including labour assessment, communication of assessment findings, and decisions regarding admission or discharge. These findings are based on women’s retrospective accounts and could not be corroborated with clinical records, provider interviews, or facility-level data, thus depicting reported experiences and perceptions rather than as evidence of deficits in individual provider competence. Being sent home in false labour appeared as an important driver for both planned and unplanned non-institutional deliveries. In the unplanned pathway, it was the immediate cause of the birth: women were told delivery would not occur for the day/s, went home, and delivered within hours. In the planned pathway, the same experience in an earlier pregnancy was what had established mistrust in providers’ clinical judgement. Having concluded based on such experiences that facility staff could not accurately judge labour, these women did not return. A single assessment that is perceived as misjudged therefore carries two costs: a birth outside a facility now, and a shift toward planned avoidance in the next pregnancy.

Non-institutional births as reported were often attended by unskilled birth attendants who lack formal training, diagnostic capacity, and access to essential medication required for safe delivery [19,38]. Traditional birth attendants (Dais) routinely act as the first point of contact for expectant mothers as they are available in close proximity of residence and families often choose them over formal healthcare facilities because they share a sense of trust, familiarity, and comfort [20,22]. Our findings further suggest that childbirth decisions were often dynamic and evolved rapidly under conditions of uncertainty and time pressure. Decision-making was often socially mediated within households and influenced by previous experiences, perceived risks, and advice from family members [20,33,38]. These dynamics reflect broader patterns of decision-making under stress, in which timely care-seeking depends not only on individual intention but also on household, social, and health-system context [12,40].

Existing evidence largely identifies fixed, cross-sectional determinants of home delivery such as income or education [5,20,31,32]. In contrast, our study underscores the importance of viewing maternal healthcare through a continuum-of-care lens. The conceptual framework of “decision-to-delivery pathway” identifies modifiable points across the antenatal, intrapartum, and postnatal periods where breakdowns in continuity of care may contribute to non-institutional deliveries. The antenatal period provides a critical opportunity to build trust, prepare families, and improve practical knowledge regarding labour recognition and timely care-seeking [36,37]. The labour phase represents a key test of health-system readiness, including accessibility and respectful maternity care [24,26]. Notably, even after home or in-transit deliveries, many families re-engaged with the formal health system for postnatal care, immunization, or management of complications, suggesting that healthcare-seeking behaviour remained largely positive despite delivery outside facilities.

From a programmatic perspective, the findings identify several actionable priorities across the spectrum of the continuum of care. During the antenatal phase, counselling on birth preparedness for labour recognition should include both women and family members, while addressing women’s concerns related to fear of surgical operations and mistrust in labour assessments [23,34]. Counselling women on labour and danger signs as part of birth preparedness may also reduce drivers of non-institutional delivery during labour by improving recognition of labour and understanding of the need for timely referral in the intrapartum phase. Additionally, promoting respectful care, ensuring labour support, and eliminating abuse and neglect are essential for positive birth experiences [16,24]. Simultaneously, the presence of a reliable transportation mechanism is dependent on the availability in the health system and geographical transport accessibility in terms of road conditions and last-mile connectivity, pointing to intersectoral policies to improve maternal and newborn outcomes [6,13,36]. Appropriate counselling in the antenatal period may also encourage women to seek care from the health system for themselves and their children, including immunisation [11,34]. The findings also highlight the importance of engaging household decision-makers alongside pregnant women, recognizing that childbirth decisions are often socially negotiated within families and communities [23,25]. For women living far from their nearest skilled birth attendants, maternity waiting homes have been proposed to improve access and birth outcomes [41].

This study has several strengths. This study provides a detailed, retrospective account of decision-making processes during labour, captured through in-depth interviews conducted in the postpartum period, offering in-depth insight into non-institutional deliveries within a setting of otherwise high institutional coverage and identifying gaps that may remain overlooked in routine programme assessments. In addition, the identification of modifiable barriers across the continuum of care enhances the practical relevance of the findings for ongoing maternal health system programmes. However, certain limitations should be considered while communicating the study results. First, the classification of delivery settings (facility or non-facility) relied on participant self-report and may have resulted in misclassification, particularly where informal or non-standard facilities were perceived as institutional deliveries. Second, the relatively small number of non-institutional deliveries within the cohort may partly reflect repeated engagement between study personnel and participants, which could have increased motivation for institutional delivery. Responses may also have been influenced by social desirability bias, which may have been compounded by participants’ pre-existing familiarity with interviewers of the ReMAPP study team, particularly with regard to experiences with healthcare services. As with all qualitative studies, recall bias may have affected reconstruction of decision-making pathways. Since the final sample interviewed was constrained by participant availability rather than confirmed through formal saturation assessment, it is possible that some less common experiences, particularly within the smaller planned pathway, may be interpreted as reflecting recurring, rather than exhaustive, themes within each pathway. Finally, the findings are context-specific to a single site and may not be fully generalizable to other settings.

Inferences related to health-system responsiveness, labour assessment, and provider practice are based on self-reporting; without triangulation with medical-record review, provider interviews, or facility/ambulance-service data, these should not be directly interpreted as clinical or health-system failures. Finally, as the interview guide included direct probes on sensitive topics such as mistreatment, informal payments, and the role of traditional birth attendants, and because the codebook was developed partly from this guide, some themes may have been elicited by targeted questioning rather than spontaneous appearance; we did not systematically distinguish spontaneously reported from probe-elicited content during coding, and this should be considered when interpreting the prominence of these themes.

## 5. Conclusions

Non-institutional births in northern India were characterized by two interconnected pathways: planned home deliveries shaped by mistrust and sociocultural preferences, and unplanned home deliveries resulting from breakdowns in labour recognition, transport, and lack of health system responsiveness. Notably, most home births in this cohort were unplanned, occurring among women who intended facility delivery and, in several cases, were sent away despite presenting in a timely manner. These findings demonstrate that improving institutional delivery coverage requires more than increasing physical access to facilities. Trust, respectful maternity care, responsiveness during labour, and practical birth preparedness may shape whether women who seek institutional care are able to access it. Strengthening continuity of care across the antenatal and intrapartum periods, alongside engagement of household decision-makers and improvements in transport solutions, may help reduce preventable non-institutional births in vulnerable populations.

## Figures and Tables

**Table 1:** Distribution of participant characteristics for planned non-institutional (n = 19) and unplanned non-institutional (n = 31) deliveries.

Variable	Category	Overall (N = 50) n (%)	Planned Non-Institutional Delivery (N = 19) n (%)	Unplanned Non-Institutional Delivery (N = 31) n (%)
Age group	18–24	31 (62)	15 (78.9)	16 (51.6)
	25 and above	19 (38)	4 (21.1)	15 (48.4)
Participant’s schooling (in completed years)	<9 years	23 (46)	10 (52.6)	13 (41.9)
	≥9 years	27 (54)	9 (47.4)	18 (58.1)
Wealth quintile *	Highest	16 (32)	6 (31.6)	10 (32.3)
	Higher	12 (24)	6 (31.6)	6 (19.4)
	Middle	9 (18)	4 (21.1)	5 (16.1)
	Lower	6 (12)	2 (10.5)	4 (12.9)
	Lowest	7 (14)	1 (5.3)	6 (19.4)
Participant’s occupation	Homemaker	48 (96)	18 (94.7)	30 (96.8)
	Working	2 (4)	1 (5.3)	1 (3.2)
Partner occupation	Skilled	28 (56)	11 (57.9)	17 (54.8)
	Unskilled labour	14 (28)	6 (31.6)	8 (25.8)
	Not working	8 (16)	2 (10.5)	6 (19.4)
Parity	Nulliparous	9 (18)	4 (21.1)	5 (16.1)
	Primiparous	20 (40)	6 (31.6)	14 (45.2)
	Multiparous	21 (42)	9 (47.4)	12 (38.7)
Previous C-section	No	48 (96)	18 (94.7)	30 (96.8)
	Yes	2 (4)	1 (5.3)	1 (3.2)
Completed antenatal care visits	<4 visits	6 (12)	2 (10.5)	4 (12.9)
	≥4 visits	44 (88)	17 (89.5)	27 (87.1)
Decision-maker for delivery place	Woman alone	4 (8)	3 (15.8)	1 (3.2)
	Husband alone	26 (52)	9 (47.4)	17 (54.8)
	Woman and husband jointly	2 (4)	0 (0)	2 (6.5)
	Mother-in-law	11 (22)	5 (26.3)	6 (19.4)
	Other family members	7 (14)	2 (10.5)	5 (16.1)

* Assets included in wealth quintile: toilet facilities, wall material, floor material, roof material, electricity, solar panels, internet access, land, mobile phone, radio, television, fridge, computer, watch, bike, motorcycle, car, animal drawn cart, plough, foam mattress, straw mattress, spring mattress, sofa, lantern, sewing machines, washing machine, blender, mosquitos net, tricycle, table, cabinets, satellite dish, DVD/CD player, air conditioner, tractor, separate sleeping room for children, land, life stock, cattle-goat, sheep, poultry, pigs, donkey, horse.

**Table 2:** Thematic analysis for planned non-institutional delivery.

Theme	Category	Level of Barrier/Driver	Code	Verbatims
Perceived mistrust	Mistrust in clinical judgement	Health system barrier	Mistrust in labour assessment	“We went to Government Hospital in a tempo, they were adamant and said that delivery will not happen today, it will happen in 2–3 days, this is what they say in the government.... Then we came back in the night and delivery happened in the morning. When they said it will happen after 2–3 days, so we did not go. (ID 31, planned)”
			Perceived unnecessary surgery or tests	“In the hospital, after seeing the ultrasound, they said that there will not be a normal delivery. It will have to be done through operation only... my blood is such that it is not clean (Rh negative)... doctor said, the test will be done from outside. When we go to a government hospital, why will we pay money outside... when there is no money, then who is so crazy to get it done from outside. (ID 13, planned)”
		Health system/individual barrier	Referral to higher facility	“I used to have pain from the beginning, I had shown at hospital as well. There was a problem so I took it there (to Dai)... here (at hospital) they do not provide any good treatment. I had taken the first baby, I don’t know they gave me an injection, then referred, they made us roam around the whole night, I went to higher facility also, they also gave me 2 injections and then said take them to another facility at night itself, we did not like it.. then I went to another hospital, then another... went to 5 places. (ID 34, planned)”
	Inadequate intrapartum support	Health system barrier	No labour support (being ignored)	“We went to the hospital, but no one gave an injection to increase the pain. I went at the time of all three children, no one gave time... I did go, but they didn’t pay attention to me, they didn’t help me in the hospital, I went twice, both times they did the same thing. (ID 19, planned)”
Institutional avoidance	Fear of medical intervention	Individual barrier	Fear of operation, stitches	“The first child was delivered at home, she is 5 years old now... this time the midwife did it... hospital staff had told to get operation, we did not want to get it done... first we went to the hospital, then when they told us about the operation, we thought we have to get it done at home itself... why would I lie... the truth is that we do not want to get the operation done... I will not get the operation at all... normal is better than cutting and all. (ID 08, planned)”
		Health system barrier	Repeated vaginal examination	“In my previous delivery, everyone, by putting their hands again and again, kept saying that the mouth (cervical opening) is not open... I have thought that I will not go, the madam, you know, she also put her hand below, checked and wrote on the paper, did not even tell me... I feel scared... that they check again and again (ID 17, planned).”
	Disrespect and neglect	Health system barrier	Stories of abuse	“Madam (doctor) shouts a lot, I am very scared… that at the time of delivery, everyone shouts, whoever goes for delivery tells... that if they cry, then they are beaten... they (staff) also hit on the cheeks, and also hit on legs... so I am scared (ID 19, planned).”
			Stories of neglect	“What did they do there, there were 2 nurses, they gave injections, then referred and she said this is beyond our understanding, then we just thought that we will go to Dai only. (ID 34, planned)”
			Long waiting hours	“There is one thing, it takes a lot of time, a lot of time is wasted,... They do not even see if anyone is having any problem, then give them number quickly. When I went for the first time in the third month, it took me the whole day. (ID 20, planned)”
Parallel informal maternity care system	Dai as trusted care provider	Community driver	Dai as First responder	“We called Dai in the morning at 7–8 a.m., she lives nearby,... Whenever I used to have pain or anything, so, I would go to her... even when there is no one home, I can go to her. (ID 21, planned)”
			Dai’s influence and assurance	“The Dais in that place is very skilled in this (conducting deliveries), whatever she says, she does, if she says she will deliver the baby within three hours, she delivers within three hours. This is a confirmed assurance (ID 29, planned).”
	Normalisation of unsafe medical practices	Community driver	Managing high-risk cases	“She (Dai) has conducted many deliveries, even in cases where the hospital said it would require an operation, she managed it and got it done as a normal delivery (ID 29, planned).”
			Unsupervised labour induction at home	“Then they also gave me an injection before delivery...they gave the injection in the back, after that the pain became more severe. (ID 21, planned)”
	Informal service packages	Community driver	Normal delivery guarantee	“She didn’t say don’t go to the hospital, it is just that we didn’t want to get the operation done and Dai said she would get it done at home, she took a guarantee. (ID 08, planned)”
			Cost negotiation	“She took around 5–6 thousand, and she took only this much just because we don’t have parents...we are poor people so she took a little less only. (ID 09, planned)”
Negotiated decision making	Household power hierarchy	Household driver	Elders and husbands influencing decisions	“My younger sister was taken to the government hospital, but no one paid attention... the nurse on duty was changing and after that the girl child died, because of this my mother told me not to go to the hospital. (ID 30, planned)”
	Denial of risk	Individual/Household driver	Will visit hospital if needed	“In the village, everyone gives birth at home, only when there is a big problem, or issue, then they visit the hospital… In the village, everyone gives birth at home, even now they are doing it… only when there is a big problem or the child is not coming, then they go (ID 13, planned).”
		Individual/Household driver	Home is a safe place	“I had already thought that I will do delivery at home, when my first daughter was born, I went hospital for no specific reason, there was no problem, and she was born just like that... so I thought delivery can happen at home, aa I dint have any problem as other women face problems. (ID 33, planned)”
	Practical constraints	Individual/Household driver	Childcare responsibility	“We need someone at home too, I had to take care of the children at home, I don’t have a mother-in-law. Earlier (in the previous pregnancy) my sister took care, when I went to have a baby. Now she too is busy working (ID 42, planned).”
			Absence of male birth companion	“At that time there was no one at home to take me to the hospital, only my mother-in-law was there, who would have taken me? nobody was there... I was having pain since 6 o’clock, I did not have the ambulance number and I did not even call, there was no one at home to take me... Father-in-law had also gone to work. (ID 21, planned)”

**Table 3:** Thematic analysis for unplanned non-institutional delivery.

Theme	Category	Level of Facilitator/Barrier	Code	Verbatims
Intention for institutional delivery and preparedness	Prior Intention	Individual/household facilitator	Plan to deliver at hospital	“All the facilities are available in the hospital. All the children in this house were born in the hospital only. Why should it be done at home, do you see any facilities at home? I don’t see any, the children do well in the hospital, as they have the facilities for everything (ID 26, unplanned).”
	Arrangement of transportation	Individual/household facilitator	Own vehicle	“Had ambulance number but the pain was severe, so quickly went to the hospital in our car. It happened on the way, as soon as we crossed the village... There was no time for the ambulance, if they had come, if I had called, then birth would have happened here (at home), that’s why I told him (husband) to come quickly, still it happened on the way. (ID 25, unplanned)”
		Health system facilitator	Arrangement of transportation by ASHA/AWW	“The Anganwadi worker, she said that whenever you feel pain, tell me and I will call an ambulance. (ID 37, unplanned)”
		Health system barrier	Delay in arrival of Ambulance	“See we wanted to go there, the ambulance was taking some time to arrive meanwhile delivery happened. The pain was for 3–4 h, it started building up in the evening, at that time pain was mild.. delivery happened around midnight. (ID 40, unplanned)”
	Perception of Hospital	Health system facilitator	Positive experiences	“All my other deliveries have been in hospitals, except for this girl at home... Both were born in government hospitals. (ID 06, unplanned)”
		Individual facilitator	Positive perception	“In today’s world, hospital is more appropriate, if you get any problem in the hospital then at least you can get it treated. (ID 16, unplanned)”
Delay in obtaining appropriate maternity care	Inability to identify labour onset	Health system barrier	Inadequate labour assessment	“We went to the hospital in the daytime, they sent me back, on Monday night, a girl was delivered at midnight. I came and lay down on the cot (bed), the child delivered there itself (ID 03, unplanned).”
		Individual barrier	Delayed recognition of labour	“We were going to the hospital, when she was going down the stairs, she felt pain twice, that’s when she delivered...the delivery took place at 11 p.m. (ID 04, unplanned)”
Physical access barriers	Restricted access to transportation	Community Barrier	Railway crossing	“We called the hospital for ambulance, the ambulance arrived, but there was a roadblock at railway crossing, he (ambulance driver) told I will not be able to come, you come by car here, but we had no one here, we told him that we won’t be able to come till there, he returned from there... the road remained closed for 2–3 months, a bridge was being built... there is no other way... the road is broken (ID 15, unplanned).”
		Community Barrier	Poor road connectivity	“Then we went to the hospital by ambulance. The ambulance wouldn’t come inside, so I had to walk all the way to the outside. I was bleeding profusely and was kept in the hospital for four days. (ID 28, unplanned)”
	Household mobility constraints	Household barrier	Mobility constraints due to stairs	“How to call the ambulance? There was no time, I was upstairs, how would they take me down, then umbilical cord also came out, when half the child came out, that’s when everyone came upstairs to see me then they brought me downstairs… At that time nobody could think of anything…. (ID 10, unplanned)”
Seeking informal birth assistance in emergent situation	Unskilled delivery support	Community driver	Dai conducting the delivery	“There is one Dai in the village... she brought a blade to cut the cord and tied it with thread... then we took the child to the hospital the next day, and they put a clip in it. (ID 04, unplanned)”
		Community driver	Household member helping during delivery	“My mother-in-law cut it, just like that, she is not trained, she tied cord with a thread. (ID 38, unplanned)”
		Community driver	Persons with no experience conducting deliveries	“We called the ambulance, the ambulance came and stopped, but before that the child was born, so the ambulance went empty, it was raining heavily….. There is a girl living in the neighborhood, her mother used to do deliveries, we called her for help, she cut the cord (ID 23, unplanned).”
Opportunities to avail health system services	Immediate care provided after birth	Health system facilitator	EMT/Doctor’s arrival after birth	“At first a girl child was born… as soon as she fell in the toilet, I quickly caught her, after catching her, her umbilical cord came out, then we called our sister, so she called the ambulance, the ambulance came and took us, girl was born at home, then a boy was born in the government hospital... (ID 07, unplanned).”
	Hospital care post-event	Individual/household facilitator	Post-delivery Care	“In the evening I went to hospital, then went to another for the injection that I had to get (for Rh negative mother) for negative blood. The injection was not available anywhere, so I went to the hospital again in the morning when I got it. The injection was not available, then I got it from our sources and then got it administered. (ID 25, unplanned)”
		Individual/household facilitator	Immunization	“The vaccination was given the next day, we took the child to there (the hospital)... it was given after 24 h. (ID 10, unplanned)”

## Data Availability

While study data were anonymised for the purposes of this study, participants may still be potentially identifiable from transcripts due to their lived experiences. The authors are not therefore sharing the data from this study in line with the ethical approval and consent granted.

## Supplementary Materials

The following supporting information can be downloaded at: https://www.mdpi.com/article/10.3390/healthcare14183126/s1.

## Abbreviations

The following abbreviations are used in this manuscript:

ReMAPP	Redefining Maternal Anemia in Pregnancy and Postpartum
LMICs	Low- and middle-income countries
JSY	Janani Suraksha Yojana
SUMAN	Surakshit Matritva Aashwasan
JSSK	Janani Shishu Suraksha Yojana
LaQshya	Labour Room Quality Improvement Initiative
IDIs	In-depth interviews
ASHA	Accredited Social Health Activist
AWW	Anganwadi Worker
