# Supplementary material for: From Decision to Delivery: A Qualitative Study of Non-Institutional Birth Pathways in Northern India
Source: Healthcare (Basel). 2026 Sep 21;14(18):3126. doi: 10.3390/healthcare14183126 (PMC13606389; doi:10.3390/healthcare14183126)
Supplement: Supplementary file 1 [file healthcare-14-03126-s001.zip › Supplementary file S2.pdf]

Article

From decision to delivery: A qualitative study of non-institutional birth pathways in Northern India

Supplementary file S2. COREQ (COnsolidated criteria for REporting Qualitative research) Checklist

Item No	Guide Questions/Description	Reported on Page #
**Domain 1: Research team and reflexivity**		
**Personal Characteristics**		
**1. Interviewer/ facilitator**	Which author/s conducted the interview or focus group?	Author Contributions
**2. Credentials**	What were the researcher's credentials? E.g., PhD, MD	Pg 4
**3. Occupation**	What was their occupation at the time of the study?	Pg 4
**4. Gender**	Was the researcher male or female?	Pg 4
**5. Experience and training**	What experience or training did the researcher have?	Pg 4
**Relationship with participants**		
**6. Relationship established**	Was a relationship established prior to study commencement?	Pg 4
**7. Participant knowledge of the interviewer**	What did the participants know about the researcher? e.g. personal goals, reasons for doing the research?	Pg 4
**8. Interviewer characteristics**	What characteristics were reported about the interviewer/facilitator? e.g. Bias, assumptions, reasons and interests in the research topic	Pg 4
**Domain 2: study design**		
**Theoretical framework**		
**9. Methodological orientation and Theory**	What methodological orientation was stated to underpin the study? e.g. grounded theory, discourse analysis, ethnography, phenomenology, content analysis	Pg 5
**Participant selection**		
**10. Sampling**	How were participants selected? e.g., purposive, convenience, consecutive, snowball	Pg 4
**11. Method of approach**	How were participants approached? e.g., face-to-face, telephone, mail, email	Pg 4
**12. Sample size**	How many participants were in the study?	Pg 6
**13. Non-participation Setting**	How many people refused to participate or dropped out? Reasons?	Pg 6

**14. Setting of data collection**	Where was the data collected? e.g., home, clinic, workplace	Pg 4
**15. Presence of nonparticipants**	Was anyone else present besides the participants and researchers?	N/a
**16. Description of sample**	What are the important characteristics of the sample? e.g. demographic data, date	Pg 3,6
**Data collection**		
**17. Interview guide**	Were questions, prompts, and guides provided by the authors? Was it pilot tested?	Pg 4
**18. Repeat interviews**	Were repeat interviews carried out? If yes, how many?	N/A
**19. Audio/visual recording**	Did the research use audio or visual recording to collect the data?	Pg 5
**20. Field notes**	Were field notes made during and/or after the interview or focus group?	Pg 5
**21. Duration**	What was the duration of the interviews or focus group?	Pg 5
**22. Data saturation**	Was data saturation discussed?	N/A
**23. Transcripts returned**	Were transcripts returned to participants for comment and/or correction?	N/A
**Domain 3: analysis and findings**		
**Data analysis**		
**24. Number of data coders**	How many data coders coded the data?	Pg 5
**25. Description of the coding tree**	Did the authors provide a description of the coding tree?	Table 2, 3
**26. Derivation of themes**	Were themes identified in advance or derived from the data?	Pg 5
**27. Software**	What software, if applicable, was used to manage the data?	Pg 5
**28. Participant checking**	Did participants provide feedback on the findings?	Pg 5
**Reporting**		
**29. Quotations presented**	Were participant quotations presented to illustrate the themes/findings? Was each quotation identified? e.g., participant number	Pg 10-14 and Table:2, 3
**30. Data and findings consistent**	Was there consistency between the data presented and the findings?	Pg 14-17
**31. Clarity of major themes**	Were major themes clearly presented in the findings?	Pg 10-14
**32. Clarity of minor themes**	Is there a description of diverse cases or a discussion of minor themes?	Pg 14-17
