# Supplementary material for: From Decision to Delivery: A Qualitative Study of Non-Institutional Birth Pathways in Northern India
Source: Healthcare (Basel). 2026 Sep 21;14(18):3126. doi: 10.3390/healthcare14183126 (PMC13606389; doi:10.3390/healthcare14183126)
Supplement: Supplementary file 1 [file healthcare-14-03126-s001.zip › Supplementary file S1.pdf]

Article

From decision to delivery: A qualitative study of non-institutional birth pathways in Northern India

Supplementary file S1: Interview guide

Pregnancy History and Care Seeking

Q1. Can you tell me about the care you received during pregnancy for your recent delivery dated _________?

Probe: ANC registration and visits, advice from ASHA/AWW/health workers, advice on place of delivery.

Decision Making about Delivery

Q2. Before labour started, where were you planning to deliver, and why?

Probe: family preference; awareness of JSY/JSSK; whether any specific facility was chosen in advance; and whether money, transportation, birth companion were planned; was she aware of signs of labour onset and danger signs; previous delivery experience.

Events around labour and birth

Q3. Can you describe the events that happened from the time labour started until the baby was born? Whether delivery occurred at home, on the way to facility, or after returning from facility

Probe: when labour started; what did you do; who was informed; whether facility/ambulance was contacted; who was involved in deciding what to do; any delay; challenges experienced such as no availability of transportation; time of travelling from your home to nearest facility; any other access issues.

Q4. Who was with you during the delivery, and what kind of support did you receive?

Probe: presence of skilled or unskilled birth attendants, Dais, Who conducted the delivery? Who cut the cord? What was used to cut and tie the umbilical cord (blade & thread/ medical kit with scissors and cord clamp); emotional support, challenges, did Dai motivate you for home birth.

Q5. Does your neighborhood have any Dais from whom people from this locality take support? For how long she has been serving? Which equipment is available with her for the delivery. Is she formally trained for conducting deliveries?

To explore: any traditional belief present behind taking the help from Dais.

Experience and perceptions of facility care

Q6. Thinking about your index pregnancy (and previous pregnancy and delivery), how were you treated?

Probe: staff behaviour (abusive behaviour, any negative comment); privacy issues; any other positive or negative experiences.

Q7. What has been your experience or perception of delivery care at health facilities?
Probe: trust in health facility; lack of staff; facility non-functional, operating hours of this facility, providers presence; lack of services; infrastructure; less/no availability of female staff; average time spent in hospital; any payments/bribes; whether procedures/examinations were explained and consent was taken; referral, admission, or discharge advice were given.

Q8. Is there any importance of institutional delivery according to you? Is it necessary? Would you suggest a pregnant mother like you to deliver at any healthcare facility?

Probe: future delivery preference

Care after delivery

Q9. How did you feel afterwards? Did you and/or the baby receive any care after the delivery and by whom?
Probe: mother/baby check-up; EMT/doctor/ASHA visit; postnatal care; vaccination; breastfeeding advice; cord care; newborn danger signs; any complications; timing of post-delivery care- immediately, within 24 hours, or later..

End of interview

Q10. How was your experience delivering at home? Do you have any other thoughts or experiences regarding home deliveries that you would like to share?

1
